# High prevalence of inadequate sitting and sleeping postures: a three-year prospective study of adolescents

**DOI:** 10.1038/s41598-017-15093-2

**Published:** 2017-11-02

**Authors:** Matias Noll, Cláudia Tarragô Candotti, Bruna Nichele da Rosa, Marja Bochehin do Valle, Arthur Antoniolli, Adriane Vieira, Jefferson Fagundes Loss

**Affiliations:** 1 Instituto Federal Goiano, Ceres, Brazil; 2 Universidade Federal do Rio Grande do Sul, Porto Alegre, Brazil

## Abstract

There is a paucity of information regarding the development of body posture during adolescence. This three-year prospective study aimed to evaluate sitting and sleeping postures among adolescents, and to determine whether these postures are associated with age and sex. We assessed 525 adolescents aged 11–16 years from the fifth to eighth grades. These adolescents were reassessed three years later. The Back Pain and Body Posture Evaluation Instrument was used to evaluate the sleeping posture and three sitting positions: sitting to write, to use a computer, and during leisure activities. Our findings indicated a low prevalence of adequate sleeping and sitting postures at baseline, with a decrease in prevalence observed after three years for all postures. These changes were similar for both sexes. Moreover, we found a strong reduction of adequate posture prevalence for younger adolescents, but the oldest adolescents demonstrated no significant differences after three years. Early, rather than late, adolescence is a critical period for establishing inadequate sitting and sleeping postures. This has implications for posture throughout adulthood; hence, interventions targeted at this age group are needed.

## Introduction

Posture is the “relative arrangement of the parts of the body” and good posture, as defined by the Posture Committee of the American Academy of Orthopedic Surgeons, is the “state of muscular and skeletal balance which protects the supporting structures of the body against injury or progressive deformity irrespective of the attitude (erect, lying, squatting, or stooping)”^1,2^. Hence, body posture is a key aspect of spine health. Maintaining the physiological curvatures of the spine is recommended as this improves load distribution to all musculoskeletal structures, is related to lower intervertebral disc pressure, and prevents musculoskeletal overload^3^. Inadequate postures, such as slump and flexed positions, can lead to back pain and musculoskeletal disorders^4–6^. Evaluating the posture of young people is essential because puberty increases the risk of spinal problems owing to hormonal, biomechanical, and behavioral factors such as altered pain perceptions, the pubertal growth spurt, and alterations in lifestyle; these factors could contribute together or apart^7^. Moreover, habits acquired during this period tend to be maintained throughout adult life^8^.

The school environment may contribute to the development of spinal problems, considering that students spend long continuous periods sitting during classes. Adopting a sitting posture may result in changes in the length and depth parameters of the spine^9^, such as thoracic hyperkyphosis and decreased lumbar lordosis^10^. The amount of time spent sitting each day, both at school—either to write (about six hours per day) or to use a computer (about two hours per day)—and outside of school needs to be evaluated in this population. Moreover, sleeping posture (about eight hours per day) adds to the total sitting time. Together, sitting and sleeping postures are adopted for >60% of each 24-h period. Therefore, it is important to evaluate these postures, especially if they are maintained for long hours without interruption and are inadequate. These factors may increase compression on the intervertebral discs leading to disc malnutrition, and may compromise the integrity of the musculoskeletal system^4,5,10–12^. In addition, a recent study performed by Minghelli *et al*.^6^ showed that the risk of developing pain symptoms was 1.77 times greater among adolescents who adopted inadequate sitting postures than among those with adequate sitting postures. Several studies have demonstrated a similar relationship between posture and spinal problems among adolescents^13–17^. Nonetheless, is important highlight the fact that these abovementioned studies were cross-sectional, and were therefore unable to establish any relationship between cause and effect.

To the best of our knowledge, only two prospective studies have approached this topic. First, Smith *et al*. investigated the alignment of the adolescent spine in a standing posture over a three-year period^18^. Second, Poussa *et al*. evaluated the development of spinal posture during the phase of peak growth^19^. In these studies, the researchers evaluated standing posture as the only measure to identify spinal alterations and/or back problems; their findings are more applicable in the clinical setting^20^. There is, thus, a need to evaluate the other postures, specifically sleeping and sitting postures (such as the posture adopted to write, to use a computer, and during leisure time) as these contribute the highest time impact daily.

Therefore, we aimed to evaluate the prevalence of adequate sitting and sleeping postures among adolescents by means of a prospective study. Given that hormonal, biomechanical, and behavioral changes occur during adolescence, it is important to determine whether sex and increasing age exert an influence over these postures. This is the first study with the intent of evaluating sitting and sleeping postures prospectively; the findings may provide health professionals with practical information applicable to adolescents, especially in the school context. Furthermore, precise information about health-related behaviors may contribute to improved engagement with health promotion activities and may enable the institution of more effective intervention programs.

## Results

**Figure 1:**
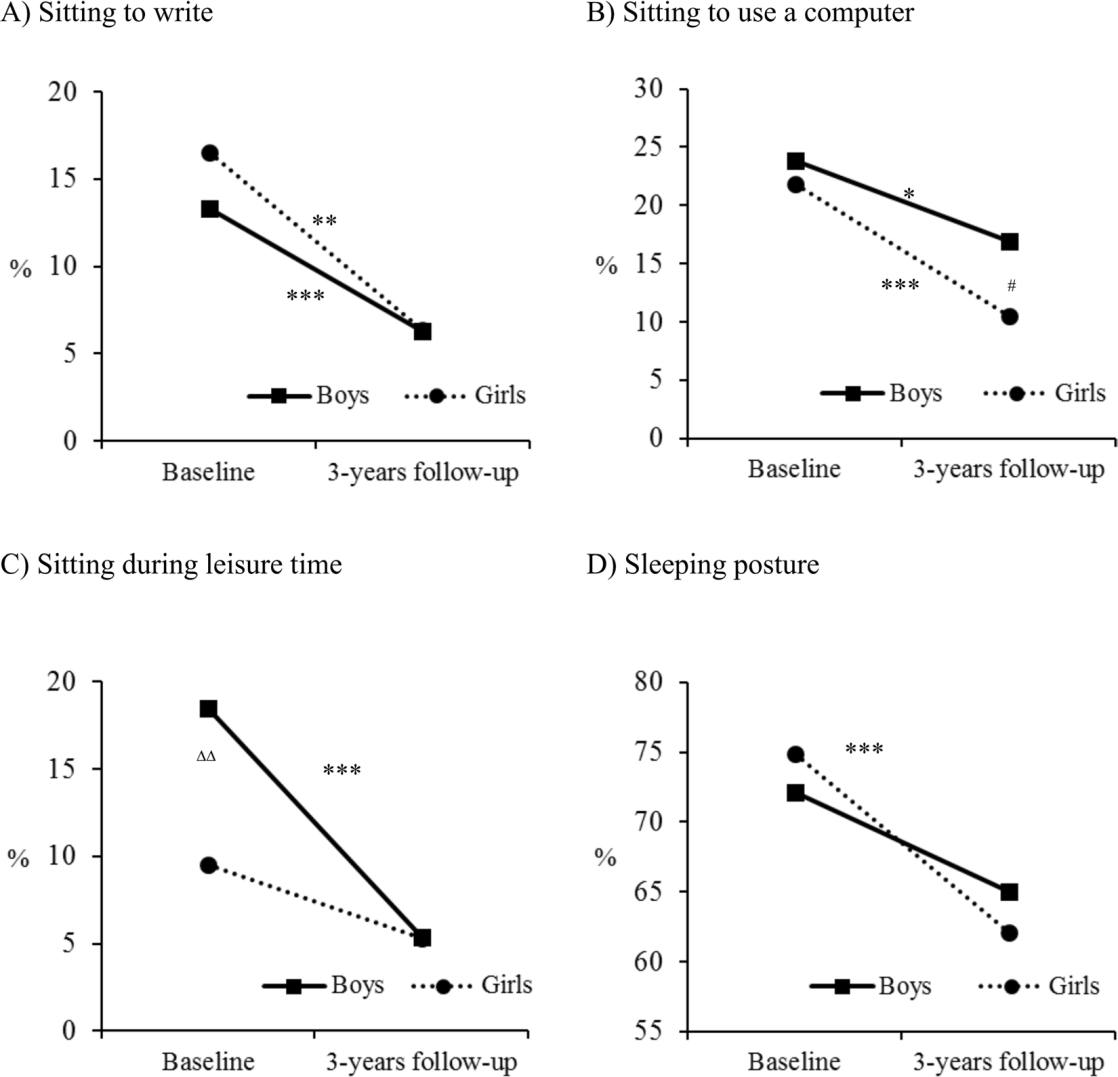
Prevalence of Adequate Sitting and Sleeping Postures at Baseline and at the Three-Year Follow-up Visit, by Sex. Significant differences between baseline and the three-year follow-up assessment (McNemar test): *p < 0.05; **p < 0.01; ***p < 0.001. Significant differences between the sexes at the three-year follow-up (χ^2^ test): ^#^ *p* < 0.05; ^##^ *p* < 0.01; ^###^ *p* < 0.001. Significant differences between the sexes at baseline (χ^2^ test): ^∆^ *p* < 0.05; ^∆∆^ *p* < 0.01; ^∆∆∆^ *p* < 0.001.

Of the 726 adolescents that participated in the baseline study, 525 (73.2%) were revaluated after three years. The prevalence of adequate posture at baseline was low for sitting positions: sitting to write (15%), sitting to use a computer (22.8%), and sitting during leisure time (13.5%). The prevalence of adequate sleeping position was higher (73.6%). Figure 1 shows that the prevalence of adequate posture for all postures was lower at the three-year follow-up assessment, with both sexes displaying the same behavior. Adequate sitting posture to write and to use a computer decreased significantly for both sexes; adequate sitting during leisure time decreased significantly for boys, only; and adequate sleeping position decreased significantly for girls, only.

**Figure 2:**
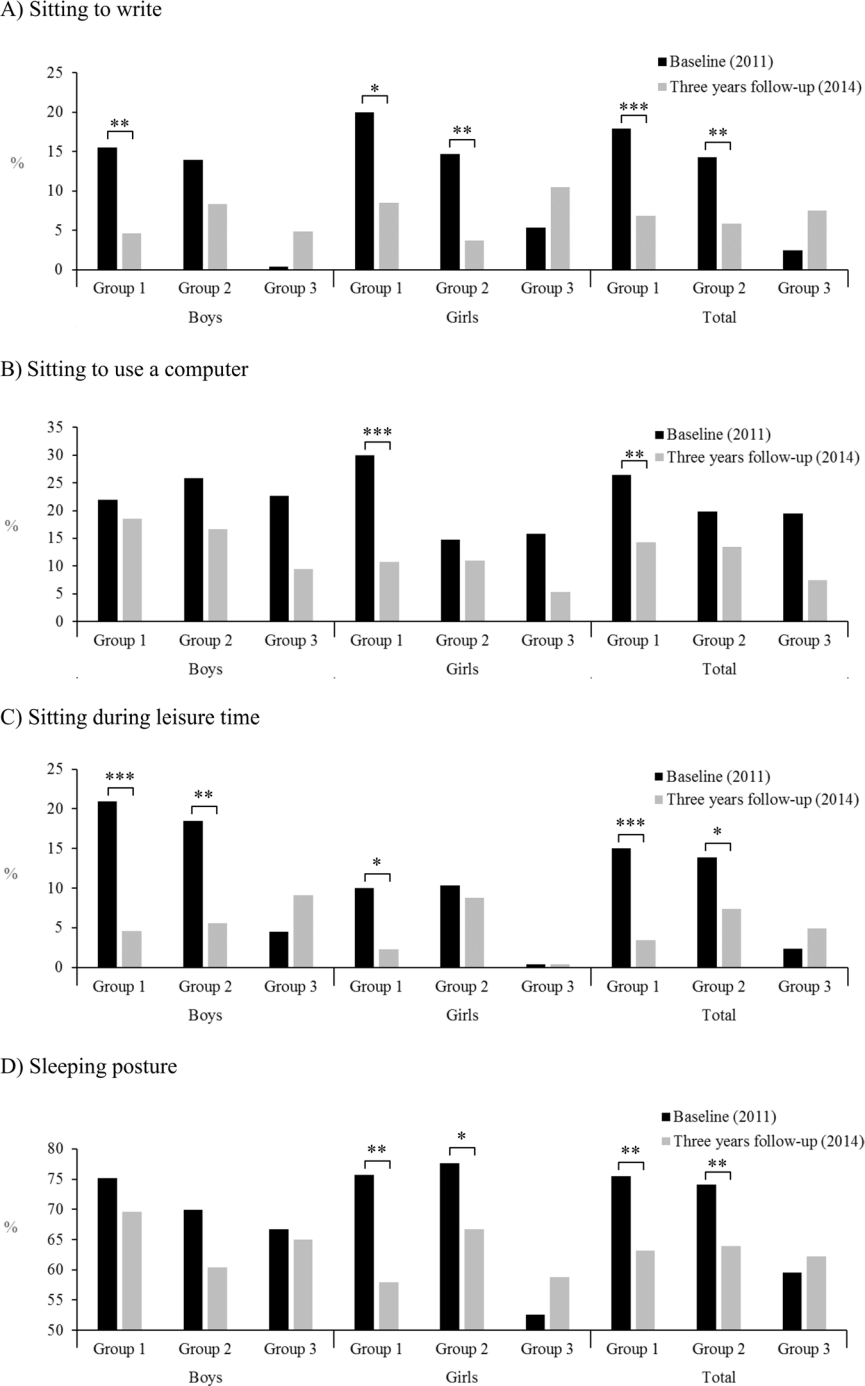
Prevalence of Adequate Sitting and Sleeping Postures at Baseline and at the Three-Year Follow-up Visit, by Age Group. Significant differences between baseline and at the three-year follow-up (McNemar test): *p < 0.05; **p < 0.01; ***p < 0.001. At baseline, “Group 1” was aged 11–12 years; “Group 2” was 13–14 years; and “Group 3” was 15–16 years.

Figure 2 shows that the decrease in prevalence of adequate posture at the three-year follow-up assessment was more marked in younger than in older adolescents, with both sexes displaying a similar pattern. The analysis of all adolescents demonstrated the prevalence of adequate posture decreased significantly for sitting to write, sitting during leisure time, and while sleeping for participants in Group 1 (aged 11 and 12 years) and Group 2 (aged 13 and 14 years). A significant decrease (from baseline to the three-year follow-up assessment) in the prevalence of adequate sitting posture to use a computer was only observed in Group 1. No significant differences in the baseline and three-year assessments were observed among the older adolescents (aged 15 and 16 years, Group 3).

## Discussion

Our main findings indicate a decrease in the prevalence of adequate sitting and sleeping postures after three years among both boys and girls. Moreover, when analyzed by subgroups of age, we found a more marked reduction in the prevalence of adequate sitting and sleeping postures among younger adolescents. In contrast, the oldest adolescents demonstrated no significant difference after three years. These findings suggest that the early period of adolescence is more critical for establishing good spinal health habits, and interventions targeted at young adolescents are needed. For example, the “I Take Care of My Spine” program, recently developed by Brzek and Plinta^10^, could be one possible intervention, since it is based on healthy activities oriented to preserve adequate body posture.

The results of the present study present some differences when compared with those of previous cross-sectional studies. Minghelli *et al*.^6^ evaluated 966 adolescents and found a higher prevalence of adequate sitting postures (~45%). Differences in the questionnaires, as well as social, cultural, and behavior factors may explain this difference. However, similar to our findings, in a study involving 1,102 adolescents, Meziat *et al.* ^15^ verified a low prevalence of adequate sitting postures while watching television (7.1%) and when using a computer (25.4%). Inadequate postures during activities of daily living are worrying because it is well established that sustained periods of adopting an inadequate postures are significantly associated with back disorders^5,6,13–15,21^; such postures may increase the force of compression on the intervertebral discs leading to disc malnutrition, may compromise the integrity of the musculoskeletal system^4^, and may predispose to fatigue and higher levels of pain^18,22^. These aspects are of concern because adolescents spend at least 60% of their day in sitting or sleeping positions. Similarly, Smith *et al*.^18^, in a three-year prospective study focused on standing postures, found that adolescents of both sexes who adopted a neutral standing posture were at lower risk of back pain than those with inadequate standing postures, supporting these findings.

Adolescence is a critical period of transition from childhood to adulthood, during which several physical and psychosocial changes take place. First, our study clearly shows a decrease in the prevalence of adequate sitting and sleeping postures after three years in boys and girls. These results are problematic because unhealthy habits related to the spine that are acquired in youth tend to remain throughout adulthood; these unhealthy habits thus urgently need to be avoided. Second, the subgroup analysis by age presented new findings, indicating that younger (rather than older) adolescents demonstrated a greater decrease in the prevalence of adequate sitting and sleeping postures after three years. In time, early adolescence overlaps with the pubertal growth spurt that is considered a particularly vulnerable period due the physical transformation of the body. This may overload and harm spinal structures, and may result in spinal disorders^7^. Unfortunately, educating adolescents about spine health, posture, and health care has been neglected or ignored by teachers and physical professionals^23^.

This perspective highlights the importance of health education programs, such as Back Schools, that aim to improve knowledge about spinal health and to educate adolescents to maintain neutral postures while engaging in activities at school and at home, and while participating in sporting activities^24^. We do need to acknowledge as a limitation to this study that assessment by means of self-reported questionnaires could lead either to overestimation or underestimation in the responses.

In conclusion, adolescents have a high prevalence of inadequate sitting and sleeping postures; this is more marked in younger than in older adolescents. This has consequences for posture throughout adulthood. Hence, we suggest the implementation of preventive and educational programs focused on improving posture and ensuring that good spinal health habits are established during the early period of adolescence.

## Methods

The study forms part of the “Brazilian Longitudinal study on Back Pain and Posture from Adolescents”. All fifth to eighth grade schools in Teutônia (*n* = 11), a municipality with 32,000 inhabitants located in the south of Brazil, were enrolled. In 2011, when the baseline study took place, a total of 1720 schoolchildren were registered in the public and private education systems.

We conducted an epidemiological exploratory and longitudinal study—we invited 736 randomly selected students from the fifth to eighth grades in the 11 schools to participate. Of these, 726 were willing to participate. Eligible students were 11–16 years of age, were enrolled in public or private schools, and were willing to participate. The exclusion criteria were missing one of the evaluation meetings and being pregnant during the study period. All students and their parents or guardians voluntarily signed an informed consent form. The present study was performed in accordance with the Helsinki Declaration and was approved by the Ethics Research Committee (number 19832).

Each participant was invited to participate in the follow-up study that was conducted three years later, in 2014. Of the baseline participants, 201 were excluded from the analysis because they did not attend the follow-up assessment for reasons including not being present on the day of the assessment; having changed school; or having stopped studying. The students were grouped into three categories according to age (Group 1: 11 and 12 years; Group 2: 13 and 14 years; Group 3: 15 and 16 years). The age and sex stratified number of participants at baseline are presented in Table 1.

**Table 1:** Frequency and Percentage of Students Evaluated at Baseline (2011), Stratified by Sex and Age.

Group	Male	Female	Total
Group 1 (11 and 12 years)	110 (45.8)	130 (45.6)	240 (45.7)
Group 2 (13 and 14 years)	108 (45)	136 (47.7)	244 (46.5)
Group 3 (15 and 16 years)	22 (9.2)	19 (6.7)	41 (7.8)
Total	240 (45.7)	285 (54.3)	525 (100)

Data are presented as *n* (%).

At baseline and at the three-year follow-up visit, participants provided answers to the Back Pain and Body Posture Evaluation Instrument (BackPEI), a self-administered, valid, and reproducible questionnaire^25^. The questionnaire addressed the following: back pain and posture characteristics; demographics; hereditary characteristics; and behavioral and sports variables. Here, we focus on the analysis of postural factors (sleeping posture; sitting posture to write; sitting posture to use a computer; and sitting posture during leisure time activities, such as conversing or reading) and demographic variables (age and sex). The BackPEI questionnaire was handed to each student individually in their classroom, and a collective explanation was given as to how the questionnaire should be answered.

The questions related to posture comprised figures showing subjects performing the activities, with a specific version for each sex to facilitate identification with the content of each question, interpretation of the question and, consequently, a more representative response. Each question had five or six options, including an “Other/I don’t know” option. Only one option was considered to be the correct way to perform each activity; the other options were grouped as inadequate for the statistical analysis. For the seated positions, the posture considered adequate was the figure showing the trunk positioned erect and neutral, preserving the natural physiological curvatures of the spine^25^. For lying down, the lateral or supine decubital positions were considered adequate^25^.

The statistical analysis was performed using IBM SPSS Statistics for Windows, Version 20.0 (Armonk, NY: IBM Corp.) Data were analyzed using descriptive and inferential statistics. We compared the data between sexes using the χ^2^ test. To perform the prospective analysis, McNemar’s test was used to compare baseline and follow-up data. A *p*-value < 0.05 was considered statistically significant.
